# Drug-coated balloons for small coronary artery disease in patients with chronic kidney disease: a pre-specified analysis of the BASKET-SMALL 2 trial

**DOI:** 10.1007/s00392-022-01995-3

**Published:** 2022-02-27

**Authors:** Felix Mahfoud, Ahmed Farah, Marc-Alexander Ohlow, Norman Mangner, Jochen Wöhrle, Sven Möbius-Winkler, Daniel Weilenmann, Gregor Leibundgut, Florim Cuculi, Nicole Gilgen, Christoph Kaiser, Marco Cattaneo, Bruno Scheller, Raban V. Jeger

**Affiliations:** 1 Universitätsklinikum des Saarlandes, Saarland University, Kirrberger Str. 1, IMED, Geb. 41.1, 66421 Homburg, Saar Germany; 2 Knappschaftskrankenhaus, Klinikum Westfalen, Dortmund, Germany; 3 SRH Wald-Klinikum, Gera, Germany; 4 Herzzentrum Dresden, Technische Universität Dresden, Dresden, Germany; 5 Department of Cardiology and Intensive Care, Medical Campus Lake Constance, Friedrichshafen, Germany; 6 University Hospital Jena, Jena, Germany; 7 Cantonal Hospital St Gallen, St Gallen, Switzerland; 8 Cantonal Hospital Baselland, Liestal, Switzerland; 9 Cantonal Hospital Luzern, Luzern, Switzerland; 10 University Hospital Basel, University of Basel, Basel, Switzerland

**Keywords:** Drug-coated balloon, Renal insufficiency, Revascularization strategies

## Abstract

**Background:**

Data on the safety and efficacy of drug-coated balloon (DCB) compared to drug-eluting stent (DES) in patients with chronic kidney disease (CKD) are scarce, particularly at long term. This pre-specified analysis aimed to investigate the 3-year efficacy and safety of DCB versus DES for small coronary artery disease (< 3 mm) according to renal function at baseline.

**Methods:**

BASKET-SMALL-2 was a large multi-center, randomized, controlled trial that tested the efficacy and safety of DCBs (*n* = 382) against DESs (*n* = 376) in small vessel disease. CKD was defined as eGFR < 60 ml/min/1.73m^2^. The primary endpoint was the composite of cardiac death, non-fatal myocardial infarction, and target vessel revascularization (MACE) during 3 years.

**Results:**

A total of 174/758 (23%) patients had CKD, out of which 91 were randomized to DCB and 83 to DES implantation. The primary efficacy outcome during 3 years was similar in both, DCB and DES patients (HR 0.98; 95%-CI 0.67–1.44; *p* = 0.937) and patients with and without CKD (HR 1.18; 95%-CI 0.76–1.83; *p* = 0.462), respectively. Rates of cardiac death and all-cause death were significantly higher among patients with CKD but not affected by treatment with DCB or DES. Major bleeding events were lower in the DCB when compared to the DES group (12 vs. 3, HR 0.26; 95%-CI 0.07–0.92; *p* = 0.037) and not influenced by presence of CKD.

**Conclusions:**

The long-term efficacy and safety of DCB was similar in patients with and without CKD. The use of DCB was associated with significantly fewer major bleeding events (NCT 01574534).

**Graphical Abstract:**

**Figure:**
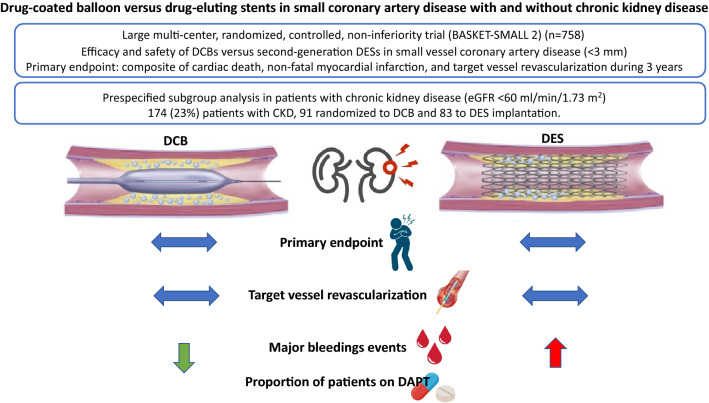
**Central Illustration**. Drug-coated balloon versus drug-eluting stents in small coronary artery disease with and without chronic kidney disease, a prespecified subgroup analysis of the BASKET-SMALL 2 trial

**Supplementary Information:**

The online version contains supplementary material available at 10.1007/s00392-022-01995-3.

## Introduction

Chronic kidney disease (CKD) and coronary artery disease (CAD) frequently coexist [1]. Cardiovascular disease remains the leading cause of morbidity and mortality in patients with CKD with a linear relationship between glomerular filtration rate and risk of cardiovascular mortality [1]. Percutaneous coronary intervention (PCI) and coronary artery bypass graft surgery (CABG) are both accepted treatment options in patients with obstructive CAD irrespective of renal function [2, 3]. However, CKD has been associated with an increased risk of procedural complications from PCI and CABG. In patients with CKD, often severe, calcified, diffuse and small vessel CAD can be found [4] which associates with higher rates of target lesion failure and pose several challenges for interventional treatment. Drug-coated balloons (DCB) are accepted treatment options for coronary in-stent restenosis and have shown to be also effective and safe in the de-novo treatment of coronary small vessel disease [5–8]. In general, patients with CKD are underrepresented in clinical trials on revascularization strategies including randomized, controlled trials investigating DCB versus drug-eluting stent (DES) implantation. Hence, the present pre-specified subgroup analysis aimed at investigating the long-term efficacy and safety of DCB versus DES for small coronary artery disease using the dataset of a large, randomized trial according to renal function at baseline.

## Methods

The Basel Kosten Effektivitäts Trial–Drug-coated balloons versus drug-eluting stents in small vessel interventions (BASKET-SMALL 2) trial was a large multi-center, randomized, controlled, non-inferiority trial that tested the efficacy and safety of DCBs against second-generation DESs in small vessel disease (< 3 mm) up to 5 years. The study was performed in 14 centers in Germany, Switzerland, and Austria in accordance with the Declaration of Helsinki and Good Clinical Practice. The study was approved by the local ethics committees and all participating patients provided written informed consent. In the present pre-specified subgroup analysis, patients were categorized by severity of chronic kidney disease according to the estimated (CKD-Epi) GFR < 60 and > 60 ml/min/1.73m^2^.

### Study design and procedures

The study details have been published elsewhere [5, 9]. In brief, patients included in the BASKET-SMALL 2 trial had an indication for PCI (either acute coronary syndrome (ACS), stable angina pectoris, or silent ischemia) of a small coronary vessel (diameter > 2.0 to < 3.0 mm). Successful predilatation of the lesion was mandatory for inclusion [10]. The major exclusion criteria were PCI of a segment of at least 3 mm in diameter in the same coronary artery, PCI of in-stent restenosis, life expectancy of < 12 months, and pregnancy.

After successful predilatation, patients were randomized (1:1) to treatment with either PCI using DES (everolimus-eluting Xience stent, Abbott Vascular, Santa Clara, CA, USA or paclitaxel-eluting Taxus Element stent, Boston Scientific, Natick, MA, USA) or paclitaxel-coated SeQuent Please balloon (B Braun Melsungen AG, Melsungen, Germany). The DCB was 4–6 mm longer than the predilatation balloon to avoid geographical mismatch and was inflated for at least 30 s at nominal pressure. In case of flow-limiting dissections or relevant residual stenosis following DCB treatment, DES implantation was recommended. The recommended dual antiplatelet therapy (DAPT) regimen consisted of acetylsalicylic acid (100 mg per day) and either clopidogrel (75 mg per day), prasugrel (10 mg per day), or ticagrelor (90 mg twice per day). The DAPT duration was 4 weeks for DCB or 6 months for DES in patients with chronic coronary syndrome (CCS), and 12 months in ACS. In patients treated with DCB and BMS, DAPT was recommended for 3 months, and in patients with DCB and DES, DAPT was recommended for 6 months. In patients requiring oral anticoagulation, current guideline recommendations were followed [11].

### Outcomes

The primary endpoint of this analysis is the composite of cardiac death, non-fatal myocardial infarction, and target vessel revascularization (TVR) during 3 years. Cardiac death was defined as any death that was not clearly of extracardiac origin, and myocardial infarction was defined according to the guidelines [12]. Secondary endpoints were all-cause death, probable or definite vessel or stent thrombosis according to the Academic Research Consortium definition, and major bleeding defined as Bleeding Academic Research Consortium type 3 to 5 bleeding [13, 14]. Net clinical benefit was defined as the combination of major adverse cardiac event and major bleeding.

### Statistical analysis

All statistical analyses were performed on the full analysis set of patients who underwent the 1- and 3-year analysis, according to the intention-to-treat principle (i.e., all patients were analyzed based on the treatment they were randomly allocated to). Categorical data are presented as frequencies and percentages (with the effect of the grouping analyzed by Pearson’s *χ*-squared test). For numerical variables, the median and interquartile range, or the mean and standard deviation are presented, as appropriate (with the effect of the grouping examined by Student's *t*-test or Wilcoxon–Mann–Whitney test, respectively). Treatment effects on the times to event within 1, 2, and 3 years were tested by Cox regressions (with study center as a stratifying factor to account for differences in baseline hazards between study centers) for the following events: all-cause death, stent thrombosis, major bleeding, net clinical benefit, and MACE, which is the composite of cardiac death, non-fatal myocardial infarction, and TVR. The Cox regressions were performed within study arm (DES/DCB) and subgroup (CKD/no-CKD), as well as globally when controlling for renal function (both models with and without interaction between treatment and renal function were fitted). No random effect was included. The assumptions of proportional hazards and homogeneity of treatment effects among study centers in the Cox models were checked (by testing the correlation of the scaled Schoenfeld residuals with time and the interaction of the stratifying factor study center with treatment in the Cox models, respectively) and are tenable. A difference in the effect of treatment would be indicated by an interaction between CKD/no-CKD and treatment arm. The analyses were conducted using the statistical software package R [15], using “two-sided” statistical tests and confidence intervals. No correction for multiple testing was applied. All analyses should be seen as exploratory, and interpretation of *p*-values should be regarded as a suggestive, continuous measure, and not as confirmatory.

## Results

Baseline characteristics for the 758 patients included in the present analysis are summarized in Table 1. From the overall population, 174 (23%) patients had an estimated GFR at baseline < 60 ml/min/1.73m^2^, and 584 (77%) had an eGFR > 60 ml/min/1.73m^2^. For those with an impaired eGFR, baseline characteristics differed from patients with a normal renal function, including older age, higher body mass index, and a greater prevalence of hypercholesterolemia, hypertension, diabetes, heart failure, and stroke/TIA, but the rates of prior MI were similar. Use of platelet inhibitors were similar, but oral anticoagulants were more frequent in those with CKD (Table 1). Out of the 174 patients with CKD, 91 were randomized to DCB and 83 to DES implantation. Baseline characteristics according to study arm (DES vs. DCB) are depicted in Supplementary Table 1.

**Table 1:** Baseline characteristics in patients with and without CKD

	All (*n* = 758)	No CKD (*n* = 584)	CKD (*n* = 174)	*p*
Age, years	67.8 (10.3)	66.1 (10.4)	73.3 (7.8)	< 0.0001
Male sex	557 (73%)	445 (76%)	112 (64%)	0.0027
Body mass index, kg/m^2^	28.29 (4.54)	28.01 (4.22)	29.21 (5.40)	0.0022
Smoking				0.0002
Current smoker	154 (21%)	137 (24%)	17 (10%)	
Former smoker	267 (36%)	203 (36%)	64 (37%)	
Hypercholesterolemia	521 (70%)	388 (67%)	133 (77%)	0.0189
Hypertension	656 (87%)	496 (85%)	160 (92%)	0.0299
Diabetes				< 0.0001
Insulin-dependent	95 (13%)	54 (9%)	41 (24%)	
Non-insulin-dependent	157 (21%)	116 (20%)	41 (24%)	
Previous myocardial infarction	293 (39%)	227 (39%)	66 (38%)	0.8930
Previous PCI	476 (63%)	359 (61%)	117 (67%)	0.1962
Previous coronary bypass graft	71 (9%)	49 (8%)	22 (13%)	0.1231
Heart failure	83 (11%)	42 (7%)	41 (24%)	< 0.0001
Stroke or transitory ischemic attack	65 (9%)	38 (7%)	28 (16%)	0.0004
Peripheral arterial obstructive disease	53 (7%)	37 (6%)	16 (9%)	0.2505
Chronic obstructive pulmonary disease	64 (8%)	41 (7%)	23 (13%)	0.0153
Coronary artery disease				0.5328
STEMI	15 (2%)	12 (2%)	3 (2%)	
NSTEMI	109 (14%)	79 (13%)	30 (17%)	
Unstable angina	90 (12%)	67 (11%)	23 (13%)	
Chronic coronary syndrome	544 (72%)	426 (73%)	118 (68%)	
Liver disease	16 (2%)	7 (1%)	9 (5%)	0.0037
Oral anticoagulation	64 (9%)	39 (7%)	25 (15%)	0.0026

Data are mean (SD), n (%), and median (IQR). Percentages calculated by excluding missing cases.

*CKD* chronic kidney disease, *PCI* percutaneous coronary intervention, *STEMI* ST elevation myocardial infarction, *NSTEMI* Non-ST elevation myocardial infarction

Procedural characteristics are depicted in Table 2 and Supplementary Table 2. There were no acute vessel occlusions in the DCB group, neither in CKD nor in the no-CKD cohort. Primary PCI results were satisfactory in 97% patients and not different between the groups (DCB vs. DES and CKD vs. no-CKD). In 7/94 CKD patients and in 26/292 patients with preserved renal function, stent implantation was required following treatment with DCB due to flow-limiting dissection or early recoil.

**Table 2:** Procedural characteristics in patients with and without CKD

	All (*n* = 758)	No CKD (*n* = 584)	CKD (*n* = 174)	*p*
Target vessel				
Left anterior descending artery	616 (81.3%)	468 (80.1%)	148 (85.1%)	0.1772
Left circumflex artery	562 (74.1%)	432 (74.0%)	130 (74.7%)	0.9227
Right coronary artery	477 (62.9%)	365 (62.5%)	112 (64.4%)	0.7201
Multivessel disease	598 (78.9%)	458 (78.4%)	140 (80.5%)	0.6372
Bifurcation lesion	51 (6.9%)	38 (6.7%)	13 (7.6%)	0.7869
Mean procedural success, n (SD)	0.97 (0.17)	0.97 (0.16)	0.97 (0.16)	0.9242
Mean number of DCB or DES, n (SD)	1.24 (0.56)	1.24 (0.57)	1.23 (0.54)	0.8115
Mean length of DCB or DES, mm (SD)	19.10 (5.43)	19.12 (5.27)	19.04 (5.93)	0.8735
Mean effective size of DCB or DES, mm (SD)	2.53 (0.27)	2.54 (0.27)	2.52 (0.24)	0.4146
Compliant balloon predilatation	558 (73.6%)	438 (75.0%)	120 (69.0%)	0.1369
Discharge medication				
DAPT	481 (63.5%)	368 (63.0%)	113 (64.9%)	0.7083
Clopidogrel	381 (50.3%)	283 (48.5%)	98 (56.3%)	0.0828
Prasugrel or ticagrelor	261 (34.4%)	212 (36.3%)	49 (28.2%)	0.0584
Duration of medication, days (median, IQR)				
Aspirin	1080 (1037, 1096)	1081 (1040, 1096)	1078 (751, 1096)	0.2103
Clopidogrel	296 (175, 376)	309 (175, 395)	215 (174, 365)	0.4695
Prasugrel or ticagrelor	361 (318, 527)	361 (318, 531)	364 (318, 442)	0.8583
DAPT	337 (183, 378)	339 (184, 387)	322 (180, 366)	0.4639
Oral anticoagulation	1056 (409, 1096)	1073 (718, 1096)	735 (350, 1090)	0.0093

*DCB* drug-coated balloon, *DES* drug-eluting stents, *CKD* chronic kidney disease, *DAPT* dual antiplatelet therapy

**Fig. 1:**
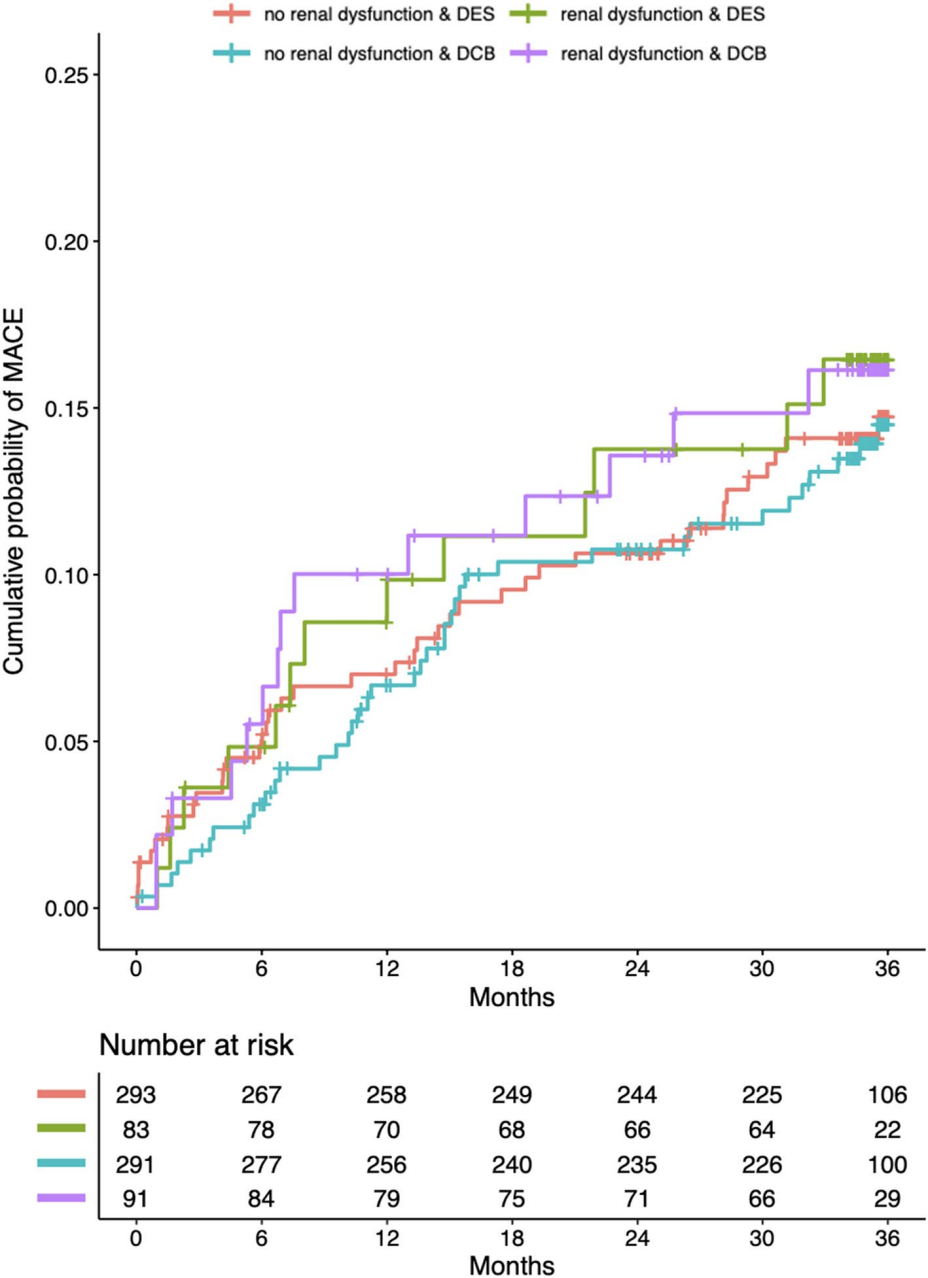
Kaplan–Meier estimates of the cumulative probabilities of MACE during 3 years in the four combinations of subgroups and study arms

A total of 349/382 (91.4%) patients in the DCB and 345/376 (91.8%) patients in the DES group completed follow-up (*p* = 0.949). The primary efficacy outcome, i.e., the composite of cardiac death, non-fatal myocardial infarction, and TVR, was similar in DCB and DES patients (HR 0.98; 95%-CI 0.67–1.44; *p* = 0.937) and not more frequent in those with CKD during 3 years of follow-up when compared to those without CKD (HR 1.18; 95%-CI 0.76–1.83; *p* = 0.462) (Fig. 1, Table 3). Rates of non-fatal myocardial infarction and TVR were not different between DCB and DES or CKD and no-CKD, respectively (Table 3), while rates of cardiac death and all-cause death were significantly higher among patients with CKD at 3 years when compared with patients without CKD (HR 2.24; 95%-CI 1.07–4.68; p = 0.032 and HR 2.59; 95%-CI 1.52–4.44; *p* = 0.001); however, this was not affected by treatment with DCB or DES.

**Table 3:** Primary and secondary outcomes

	Variable	1-year HR [95% CI]	2-year HR [95% CI]	3-year HR [95% CI]
Primary endpoint	Study arm: DCB vs DES Subgroup: CKD vs No CKD	0.96 [0.57, 1.63], *p = *0.885 1.47 [0.83, 2.62], *p = *0.186	1.00 [0.65, 1.54], *p = *0.988 1.29 [0.80, 2.10], *p = *0.301	0.98 [0.67, 1.44], *p = *0.937 1.18 [0.76, 1.83], *p = *0.462
	Study arm: DCB vs DES Subgroup: CKD vs No CKD Interaction: CKD and DCB	0.96 [0.56, 1.63], *p = *0.880 1.47 [0.83, 2.62], *p = *0.187 1.03 [0.33, 3.26], *p = *0.956	1.01 [0.65, 1.55], *p = *0.976 1.29 [0.80, 2.10], *p = *0.298 0.93 [0.35, 2.45], *p = *0.885	0.99 [0.67, 1.44], *p = *0.941 1.18 [0.76, 1.83], *p = *0.461 0.97 [0.40, 2.33], *p = *0.938
Cardiac death	Study arm: DCB vs DES Subgroup: CKD vs No CKD	2.28 [0.80, 6.49], *p = *0.121 2.25 [0.84, 5.99], *p = *0.105	1.51 [0.65, 3.49], *p = *0.337 2.09 [0.90, 4.86], *p = *0.088	1.26 [0.61, 2.61], *p = *0.524 2.24 [1.07, 4.68], *p = *0.032
	Study arm: DCB vs DES Subgroup: CKD vs No CKD Interaction: CKD and DCB	2.00 [0.67, 5.94], *p = *0.214 1.65 [0.48, 5.68], *p = *0.428 3.66 [0.31, 43.37], *p = *0.304	1.44 [0.60, 3.46], *p = *0.414 2.01 [0.84, 4.84], *p = *0.118 1.36 [0.24, 7.80], *p = *0.733	1.27 [0.59, 2.72], *p = *0.546 2.24 [1.06, 4.71], *p = *0.034 1.00 [0.23, 4.38], *p = *0.996
Non-fatal myocardial infarction	Study arm: DCB vs DES Subgroup: CKD vs No CKD	0.45 [0.17, 1.18], *p = *0.103 1.74 [0.66, 4.59], *p = *0.265	0.74 [0.37, 1.47], *p = *0.390 1.00 [0.43, 2.31], *p = *0.999	0.82 [0.45, 1.51], *p = *0.528 0.86 [0.40, 1.87], *p = *0.711
	Study arm: DCB vs DES Subgroup: CKD vs No CKD Interaction: CKD and DCB	0.45 [0.17, 1.21], *p = *0.113 1.72 [0.61, 4.85], *p = *0.307 0.94 [0.12, 7.47], *p = *0.950	0.74 [0.37, 1.47], *p = *0.388 0.99 [0.43, 2.31], *p = *0.987 0.91 [0.17, 4.96], *p = *0.915	0.83 [0.45, 1.52], *p = *0.541 0.87 [0.40, 1.88], *p = *0.718 1.15 [0.24, 5.41], *p = *0.859
Target vessel revascularization	Study arm: DCB vs DES Subgroup: CKD vs No CKD	0.75 [0.36, 1.55], *p = *0.437 0.82 [0.33, 2.02], *p = *0.671	0.89 [0.51, 1.57], *p = *0.696 0.84 [0.42, 1.69], *p = *0.619	0.95 [0,58, 1.57], *p = *0.854 0.72 [0.37, 1.39], *p = *0.326
	Study arm: DCB vs DES Subgroup: CKD vs No CKD Interaction: CKD and DCB	0.68 [0.31, 1.49], *p = *0.336 0.61 [0.19, 1.94], *p = *0.401 0.19 [0.02, 1.91], *p = *0.159	0.86 [0.49, 1.54], *p = *0.618 0.76 [0.36, 1.62], *p = *0.483 0.35 [0.08, 1.58], *p = *0.174	0.92 [0.55, 1.53], *p = *0.740 0.69 [0.35, 1.36], *p = *0.285 0.48 [0.12, 1.87], *p = *0.291
All-cause death	Study arm: DCB vs DES Subgroup: CKD vs No CKD	1.82 [0.81, 4.09], *p = *0.146 2.25 [1.02, 4.94], *p = *0.044	1.25 [0.67, 2.37], *p = *0.483 3.09 [1.64, 5.83], *p* < 0.001	1.02 [0,60, 1.73], *p = *0.948 2.59 [1.52, 4.44], *p = *0.001
	Study arm: DCB vs DES Subgroup: CKD vs No CKD Interaction: CKD and DCB	1.54 [0.65, 3.63], *p = *0.325 1.78 [0.71, 4.47], *p = *0.222 3.58 [0.57, 22.65], *p = *0.175	1.13 [0.55, 2.29], *p = *0.744 3.01 [1.58, 5.74], *p = *0.001 1.56 [0.43, 5.62], *p = *0.500	0.94 [0.53, 1.67], *p = *0.822 2.58 [1.50, 4.44], *p = *0.001 1.51 [0.51, 4.45], *p = *0.455

Cox regression with the corresponding hazard ratios and 95% CIs stratified by study center and adjusted for renal function (with and without interaction with treatment).

The primary endpoint was the composite of cardiac death, non-fatal myocardial infarction, and target vessel revascularization

*DCB* drug-coated balloon, *DES* drug-eluting stents, *CKD* chronic kidney disease

In DCB compared with DES treated patients with CKD, the median duration of DAPT was not different (323 vs. 314 days; *p* = 0.8402) (Supplementary Table 2). However, the proportion of CKD patients on DAPT (74% vs. 57%, *p* = 0.0358) and/or clopidogrel (69% vs. 45%, *p* = 0.0028) was significantly lower in the DCB compared with DES group, respectively, as mandated by the protocol (Table 4).

**Table 4:** Antithrombotic regimen in patients with CKD according to treatment group

	DES	DCB	p
Medication			
DAPT	61 (74%)	52 (57%)	0.0358
Clopidogrel	57 (69%)	41 (45%)	0.0028
Prasugrel or ticagrelor	20 (24%)	29 (32%)	0.3322
Duration of medication, days (median, IQR)			
DAPT	314 (183, 365)	323 (177, 368)	0.8402
Aspirin	1080 (968, 1096)	1078 (741, 1096)	0.7848
Clopidogrel	312 (179, 365)	211 (142, 368)	0.2930
Prasugrel or ticagrelor	355 (194, 816)	364 (324, 417)	0.6325

*DCB* drug-coated balloon, *DES* drug-eluting stents, *DAPT* dual antiplatelet therapy, *IQR* interquartile range

At 3-years, the number of probable and definite vessel or stent thrombosis in patients without CKD was 6 for DES and 2 for DCB (HR 0.32; 95%-CI 0.06–1.60; *p* = 0.167), respectively. There were no such events documented in patients with CKD.

**Fig. 2:**
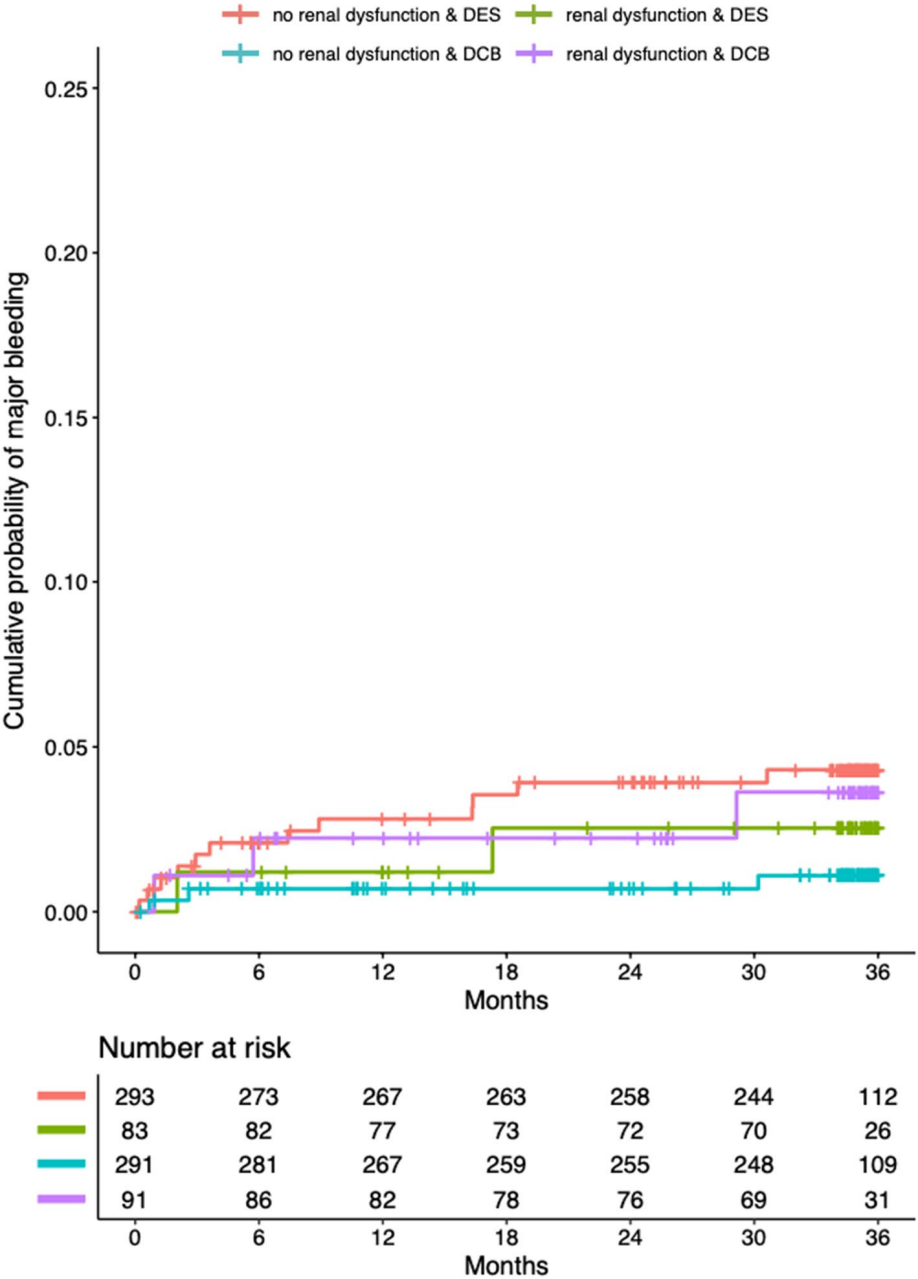
Kaplan–Meier estimates of the cumulative probabilities of major bleeding during 3 years in the four combinations of subgroups and study arms

Overall, the number of major bleeding events at 3 years were lower in patients treated with DCB when compared with DES (12 vs. 3, HR 0.26; 95%-CI 0.07–0.92; *p* = 0.037) (Fig. 2). The net clinical benefit did not differ between the groups.

## Discussion

The major findings from this pre-specified analysis of the BASKET-SMALL 2 trial are: (i) the long-term safety and efficacy of DCB was similar in patients with and without CKD, (ii) rates of both cardiac and all-cause death were significantly higher among patients with CKD compared with patients with normal renal function, and (iii) small coronary artery treatment with DCB was associated with fewer major bleeding events (Central illustration). To our knowledge, this is the first pre-specified analysis of a randomized, controlled trial with long-term follow-up focusing on DCB use in patients with CKD.

Despite the availability of various interventional techniques, the treatment of small coronary artery disease remains challenging mainly because of recoil after plain old balloon angioplasty (POBA) and neointimal hyperplasia after stent implantation [16]. DCBs combine the principle of angioplasty alone with local drug delivery using highly lipophilic drugs [17]. Paclitaxel-coated balloons have shown favorable results in the treatment of in-stent restenosis and native vessel disease [5–7, 18]. A recently published meta-analysis comprising 4590 patients confirmed the safety of these devices and indicated a trend toward lower mortality following DCB when compared with control treatments (consisting of DES, BMS and POBA [7]). The randomized, controlled BASKET-SMALL 2 trial documented non-inferiority of DCB over DES treatment in patients with small coronary artery disease over 3 years [5, 6]. It was uncertain, however, whether the outcomes of DCB treatment are affected by the presence of CKD. Out of the 758 patients included in the trial, 23% had CKD with an eGFR < 60 ml/min/1.73m^2^. The rate of major cardiovascular events at 3 years was similar in patients with CKD after treatment with DCB or DES, confirming the maintained efficacy and safety of DCB in these high-risk patients with small vessel coronary artery disease.

Chronic kidney disease is increasing worldwide and associates with pronounced risk for cardiovascular events. Indeed, 50% of all patients with CKD in stage 4–5 have cardiovascular disease, and cardiovascular mortality accounts for up to 50% of all deaths in this cohort [19]. One of the most frequent comorbidities in CKD is coronary artery disease, which represents the most common cause of morbidity and mortality in these patients [3]. Revascularization strategies in CKD include PCI and CABG surgery. Both approaches have been associated with increased risk of complications (including renal injury) and higher event rates as well as impaired success rates [2, 20]. In a pooled patient-level analysis on 12,426 patients undergoing PCI using second-generation DES, a total of 2927 patients (23.6%) had CKD (defined as eGFR < 60 ml/min/1.73m^2^). CKD patients showed significantly higher risk of target lesion failure (adjusted HR: 1.50; 95%-CI 1.21–1.86) compared with patients with preserved renal function [21]. These findings were recently supported by a study investigating 19,475 patients, including 1466 patients with CKD undergoing latest-generation abluminal sirolimus-eluting stent implantation [4]. In this study, patients with CKD had a higher risk of target lesion failure (odds ratio (OR): 2.51; 95%-CI 2.04–3.08), target vessel failure (OR: 2.44; 95%-CI 2.01–2.96), and major adverse cardiovascular events (OR: 2.34; 95%-CI 1.93–2.83, *p* < 0.0001) at 1 year when compared with patient with persevered renal function. Herein, the rates of MACE were generally low and not significantly higher in patients with CKD, but cardiac death and all-cause death were more than twofold higher among patients with CKD at 3 years (HR 2.24; 95%-CI 1.07–4.68; *p* = 0.032 and HR 2.59; 95%-CI 1.52–4.44; *p* = 0.001).

Chronic kidney disease predicts, unlike other risk factors, ischemic and bleedings complications after PCI, both of which contribute to increased morbidity and mortality [22, 23]. The choice and duration of antiplatelet therapy in CKD patients undergoing PCI is therefore often challenging. A study on 5018 PCI patients (839 with CKD) found 2–3 fold higher risks for death, ischemic and bleeding complications in CKD when compared with preserved renal function [24]. Interestingly, DAPT discontinuation during the first year after PCI was significantly more likely to occur among CKD patients, which may reflect clinical tendencies to avoid bleeding complications [24]. A revascularization strategy that can be used with a shortened course of DAPT , e.g., 4 weeks, to minimize the risk of bleeding may thus be preferable in CKD patients. Herein, the proportion of CKD patients discharged on DAPT was significantly lower for DCB when compared with DES patients (74% vs. 57%, *p* = 0.0358). Importantly, this was not associated with higher rates of ischemic complications. The major bleeding events were generally low, yet numerically lower in patients treated with DCB.

### Limitations

Although this was a pre-specified subgroup analysis, this trial was neither designed nor powered to detect differences between patients with and without CKD. Patients were not randomized according to presence of CKD at baseline. The analysis used baseline renal function and does not take account of patients with a deterioration in renal function during the course of the study. There is no randomized, controlled trial available which assessed the optimal DAPT duration following DCB treatment. The duration of DAPT however, was mandated by the protocol and in general shorter in patients following DCB treatment. Despite the lower proportion of patients on DAPT, there were no differences in ischemic events between DCB and DES treatment. There were a limited number of patients with advanced CKD and thus the findings may not generalize to patients requiring dialysis. The study was not powered to assess whether endpoints, such as stent thrombosis or all-cause death, were differentially affected by treatment with DCB or DES or the type and duration of DAPT. Consequently, the present findings should be regarded as hypothesis-generating and require confirmation by a dedicated trial investigating the performance of DCB in patients with various stages of CKD.

## Conclusions

In this pre-specified analysis of a randomized, controlled trial focusing on DCB use in CKD patients with small coronary artery disease, the long-term efficacy and safety of DCB was similar in patients with and without CKD. While rates of both cardiac and all-cause death were higher among patients with CKD compared with patients with normal renal function, the use of DCB was associated with fewer bleeding events. In patients with CKD, revascularization with DCB may represent an alternative to established strategies using metallic implants after successful predilatation. Further studies in larger cohorts of patients with CKD are required before definite conclusions can be drawn.

## Supplementary Information

Below is the link to the electronic supplementary material.

Supplementary file1 (DOCX 25 KB)
