# Supplementary material for: Drug-coated balloons for small coronary artery disease in patients with chronic kidney disease: a pre-specified analysis of the BASKET-SMALL 2 trial
Source: Clin Res Cardiol. 2022 Feb 27;111(7):806–15. doi: 10.1007/s00392-022-01995-3 (PMC9242956; doi:10.1007/s00392-022-01995-3)
Supplement: Supplementary file 1 — Supplementary file1 (DOCX 25 KB) [file 392_2022_1995_MOESM1_ESM.docx]

# Supplemental Table 1. Baseline characteristics in patients with CKD according to study arm.

|  | **All (n=174)** | **DES (n=83)** | **DCB (n=91)** | **p** |
| --- | --- | --- | --- | --- |
| Age, years | 73.3 (7.8) | 73.7 (7.2) | 73.0 (8.4) | 0.5816 |
| Male sex | 112 (64%) | 46 (55%) | 66 (73%) | 0.0282 |
| Body mass index, kg/m^2^ | 29.21 (5.4) | 29.38 (5.4) | 29.06 (5.4) | 0.6943 |
| Smoking |  |  |  | 0.6534 |
| Current smoker | 17 (10%) | 7 (9%) | 10 (11%) |  |
| Former smoker | 64 (37%) | 29 (35%) | 35 (39.3%) |  |
| Hypercholesterolemia | 133 (77%) | 61 (74%) | 72 (79%) | 0.5780 |
| Hypertension | 160 (92%) | 77 (93%) | 83 (91%) | 0.9208 |
| Diabetes | 41 (24%) | 23 (28%) | 18 (20%) | 0.1275 |
| Previous myocardial infarction | 66 (38%) | 27 (33%) | 39 (43%) | 0.2128 |
| Previous PCI | 117 (67%) | 56 (68%) | 61 (67%) | 1.0000 |
| Previous coronary bypass graft | 22 (13%) | 8 (10%) | 14 (15%) | 0.3624 |
| Heart failure | 41 (24%) | 16 (19%) | 25 (28%) | 0.2742 |
| Stroke or transitory ischemic attack | 28 (16%) | 13 (16%) | 15 (16%) | 0.7787 |
| Peripheral arterial obstructive disease | 16 (9%) | 6 (7%) | 10 (11%) | 0.5689 |
| Chronic obstructive pulmonary disease | 23 (13%) | 10 (12%) | 13 (14%) | 0.8327 |
| Coronary artery disease |  |  |  | 0.9016 |
| STEMI | 3 (2%) | 1 (1%) | 2 (2%) |  |
| NSTEMI | 30 (17%) | 13 (16%) | 17 (19%) |  |
| Unstable angina | 23 (13%) | 11 (13%) | 12 (13%) |  |
| Chronic coronary syndrome | 118 (68%) | 58 (70%) | 60 (66%) |  |
| Liver disease | 9 (5%) | 5 (6%) | 4 (4%) | 0.8872 |
| Oral anticoagulation | 25 (15%) | 8 (10%) | 17 (19%) | 0.1230 |

Data are mean (SD), n (%), and median (IQR). Percentages calculated by excluding missing cases. CKD: chronic kidney disease; PCI: percutaneous coronary intervention; STEMI: ST elevation myocardial infarction; NSTEMI: Non-ST elevation myocardial infarction;

**Supplemental Table 2.** Procedural characteristics in patients with CKD according to study arm.

|  | **All (n=174)** | **DES (n=83)** | **DCB (n=91)** | **p** |
| --- | --- | --- | --- | --- |
| Target vessel |  |  |  |  |
| Left anterior descending artery | 148 (85%) | 70 (84%) | 78 (86%) | 0.9668 |
| Left circumflex artery | 130 (75%) | 57 (69%) | 73 (80%) | 0.1152 |
| Right coronary artery | 112 (64%) | 56 (68%) | 56 (62%) | 0.5108 |
| Multivessel disease | 140 (81%) | 63 (76%) | 77 (85%) | 0.2091 |
| Bifurcation lesion | 13 (8%) | 9 (11%) | 4 (4%) | 0.1684 |
| Mean procedural success, n (SD) | 0.97 (0.17) | 0.97 (0.16) | 0.97 (0.18) | 0.9132 |
| Mean number of DCB or DES, n (SD) | 1.23 (0.54) | 1.27 (0.54) | 1.20 (0.54) | 0.4151 |
| Mean length of DCB or DES, n (SD) | 19.04 (5.93) | 17.60 (6.63) | 20.36 (4.90) | 0.0020 |
| Mean effective size of DCB or DES, mm (SD) | 2.52 (0.24) | 2.57 (0.25) | 2.48 (0.23) | 0.0161 |
| Compliant balloon predilatation | 120 (69%) | 59 (71%) | 61 (67%) | 0.6797 |
| Discharge medication |  |  |  |  |
| DAPT | 113 (65%) | 61 (74%) | 52 (57%) | 0.0358 |
| Clopidogrel | 98 (56%) | 57 (69%) | 41 (45%) | 0.0028 |
| Prasugrel or ticagrelor | 49 (28%) | 20 (24%) | 29 (32%) | 0.3322 |
| Duration of medication, days (median, IQR) |  |  |  |  |
| Aspirin | 1078 (751, 1096) | 1080 (968, 1096) | 1078 (741, 1096) | 0.7848 |
| Clopidogrel | 215 (174, 366) | 312 (179, 365) | 211 (142, 368) | 0.2930 |
| Prasugrel or ticagrelor | 360 (318, 586) | 356 (194, 1056) | 364 (333, 385) | 0.8685 |
| DAPT | 322 (180, 366) | 314 (183, 365) | 323 (177, 368) | 0.8402 |
| Oral anticoagulation | 735 (350, 1090) | 711 (359, 1085) | 889 (342, 1093) | 0.7661 |

DCB: drug-coated balloon, DES: drug-eluting stents, CKD: chronic kidney disease; DAPT: dual antiplatelet therapy;
